# Cold Agglutinin Syndrome as the Initial Presentation of Acute Myeloid Leukemia: A Case Report

**DOI:** 10.1002/ccr3.71061

**Published:** 2025-09-29

**Authors:** Mohsen Vakili Sadeghi, Zeinab Vosough, Davoud Jahansouz

**Affiliations:** ^1^ Cancer Research Center, Health Research Institute Babol University of Medical Sciences Babol Iran; ^2^ Department of Pathology Babol University of Medical Sciences Babol Iran

**Keywords:** acute myeloid leukemia, agglutinin, autoimmune hemolytic anemia, cold agglutinin disease

## Abstract

Cold agglutinin‐mediated autoimmune hemolytic anemia, when secondary to viral and bacterial infections or, rarely, malignancy, is called cold agglutinin syndrome. This is the first case in which acute myeloid leukemia (AML) was the underlying cause of this syndrome. Here, we present a 64‐year‐old man with weight loss, jaundice, anemia, and a recent episode of red urine. The blood drawn from the patient clotted swiftly, and then the cold agglutinin test became positive. Bone marrow specimen microscopy revealed monocytic AML. Standard treatment resulted in clinical improvement, although he later faced progression to hyperleukocytosis and liver failure, which led to his death. This case emphasizes the necessity of maintaining a level of suspicion for underlying hematologic malignancies in individuals with secondary cold agglutinin‐mediated hemolytic anemia. This is the first documented case of cold agglutinin syndrome related to AML, contributing to the growing knowledge of the connection between autoimmune hemolytic anemia and hematologic cancers.

**Summary**

- Cold agglutinin‐mediated autoimmune hemolytic anemia can be primary, called cold agglutinin disease (CAD), or secondary to an underlying disease, called cold‐agglutinin syndrome (CAS).
- Secondary causes are viral and bacterial infections or, rarely, a malignancy.
- This is the first case in which acute myeloid leukemia was the underlying cause of CAS and emphasizes that hemolysis caused by cold agglutinin requires a full workup, including bone marrow biopsy.

## 1 Introduction

Cold agglutinin (CA)‐mediated autoimmune hemolytic anemia (AIHA) is a rare form of anemia, accounting for 15%–25% of AIHA cases. This condition may arise as a primary disorder, known as cold agglutinin disease (CAD), or it can be secondary to other diseases, referred to as cold agglutinin syndrome (CAS) [1]. Previous studies have shown that CAD differs from Waldenström macroglobulinemia, lymphoplasmacytic lymphoma, or marginal zone lymphoma [2, 3].

CAS, which occurs less frequently than CAD, is linked to viral or bacterial infections and, on rare occasions, malignancies. Known pathogens associated with this condition include Mycoplasma pneumonia and Epstein–Barr virus [4, 5]. Additionally, varicella virus [6], influenza [7], Citrobacter [8], and even the coronavirus disease 2019 (COVID‐19) [9] have been reported.

Malignancies that may be connected with CAS include bladder urothelial carcinoma [10], uterine sarcoma [11], hepatocellular carcinoma [12], and even metastatic melanoma [13].

In most cases, CAs are monoclonal IgM with kappa light chains, although they can also be polyclonal IgM, lambda light chains, or, in rare instances, another type of immunoglobulin. Polyclonal IgM CAs typically emerge in post‐infectious situations [2]. IgM CAs attach to the I antigens present on red blood cell (RBC) membranes at temperatures lower than the body's core temperature. The ideal temperature for this binding is 3°C–4°C, but CA thermal amplitudes can reach up to 28°C–30°C or even higher, leading to RBC agglutination. When RBCs move to the peripheral areas of the body, such as the ears, nose, fingers, and toes, IgM effectively activates the complement system and initiates the classical pathway due to the large pentaglobin structure of IgM. The C3b components of the complement coat the RBC membranes. When the RBCs return to warmer core temperature regions, IgM detaches from the RBCs, and the liver and spleen's reticuloendothelial cells remove the C3d‐coated RBCs from circulation; consequently, extravascular hemolysis is the primary mechanism of hemolysis in CA‐mediated hemolytic anemia. A small percentage of patients may also experience intravascular hemolysis.

## 2 Case History/Examination

A 64‐year‐old man was admitted to the emergency room with weakness and jaundice in mid‐autumn. He had been experiencing anorexia for 6 months, with a 15 kg weight loss. One week before admission, the patient had an episode of unexplained fever, anorexia, and passed red urine for 2 days. He became icteric and visited our center because of anemia and hyperbilirubinemia detected in his laboratory tests. There was a history of recent dyspnea and dizziness. He recalled a recent laboratory contact for blood resampling due to clotting, and his blood samples were kept in 37°C until serum separation. He had no history of pain or color change on his fingertips. He was living in a humid, temperate climate.

The patient's previous medical history included type 2 diabetes mellitus from 6 years ago. The family, social, and genetic history did not contain anything significant. He was not a smoker or alcohol user at all. Surgical history included renal stone surgery, benign prostatic hyperplasia surgery, and a recent cataract surgery (9 months ago) with no major complications.

In the physical examination, his vital signs were stable, with a 36.9°C mouth temperature and a blood pressure of 123/80 mmHg. He weighed 74 kg at presentation and was pale and jaundiced upon inspection, with no rash. Fingertips, toenails, ears, and nose appeared normal with no signs of discoloration. No heart murmur was detected. Also, no hepato‐splenomegaly or lymphadenopathy was found on palpation.

Pre‐admission laboratory studies showed a white blood cell (WBC) count of 17,940 μL, a red blood cell (RBC) count of 2.06 million μL, a hemoglobin (Hb) level of 6.2 g/dL (mean corpuscular volume = 108 femtoliter), and a platelet count of 75,000 μL. Total bilirubin was 11.4 mg/dL, direct bilirubin was 3.6 mg/dL, aspartate transaminase (AST) was 58 U/L, and alanine transaminase (ALT) was 72 U/L.

**FIGURE 1:**
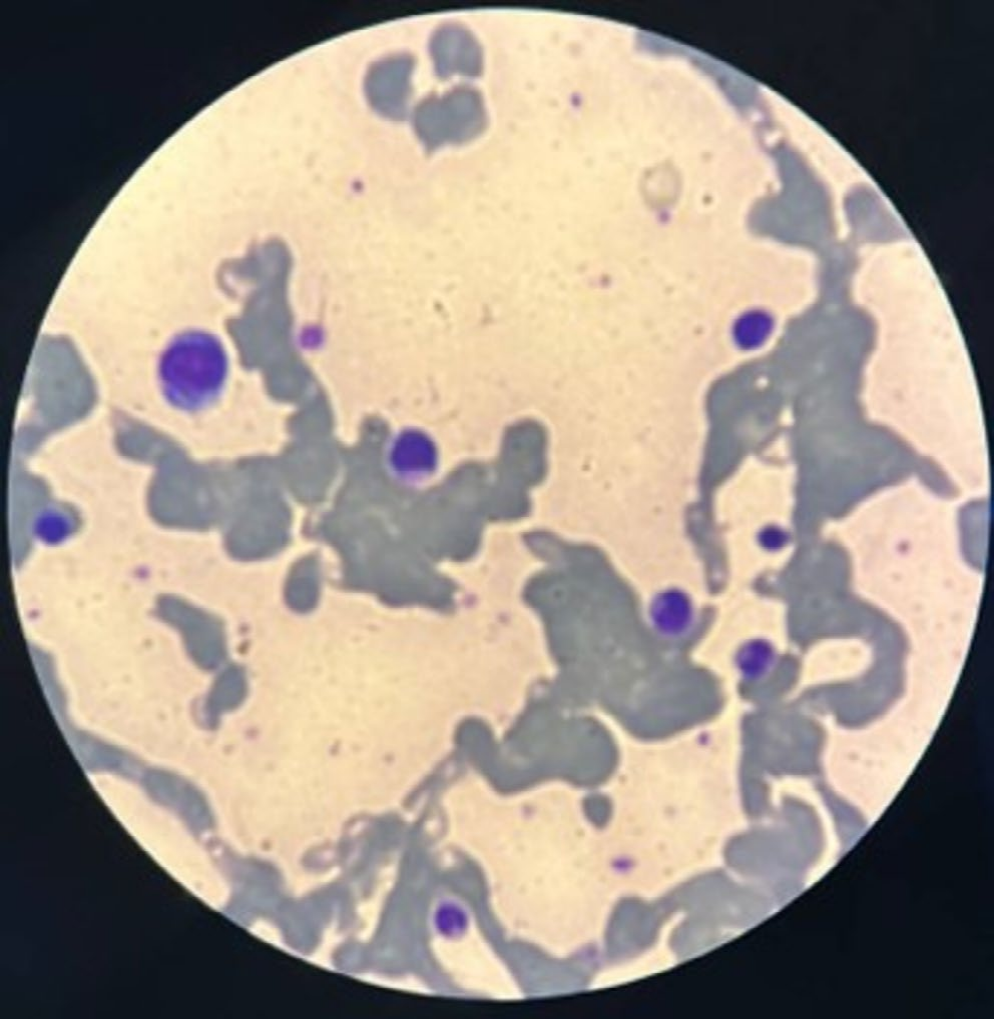
The patient's peripheral blood smear demonstrates marked red blood cell agglutination, which is consistent with cold agglutinin disease. Several myeloblasts with a high nuclear‐cytoplasmic ratio and prominent nucleoli are also seen. Wright‐Giemsa stain, magnification: 400×.

On the first day of hospitalization, a repeated peripheral blood smear examination showed RBC clumps similar to grape clusters (Figure 1); WBC differentiation was 38% lymphocytes, 34% neutrophils, 12% monocytes, 7% myelocytes, 4% metamyelocytes, and 3% eosinophils. Serum lactate dehydrogenase (LDH) level was 1980 U/L, total bilirubin was 5.2 mg/dL, and direct bilirubin was 2.3 mg/dL. The CA titer was requested, which was positive at 1/64, but the direct agglutination test was negative. Serum protein electrophoresis revealed polyclonal hypergammaglobulinemia (20.8% [reference value up to 18.8%]). Moreover, his Hb was 7.5 g/dL. One unit of packed RBCs was transfused with a warmer set, which was repeated 2 days later. Based on a presumptive diagnosis of idiopathic CAD and a good response to rituximab in this disease [14], Rituximab was prescribed at a single dose of 375 mg per square meter.

### 2.1 Differential Diagnosis

Infections (bacterial and viral) and blood/solid malignancies were thought to be the most likely underlying disease.

## 3 Conclusion and Results

**FIGURE 2:**
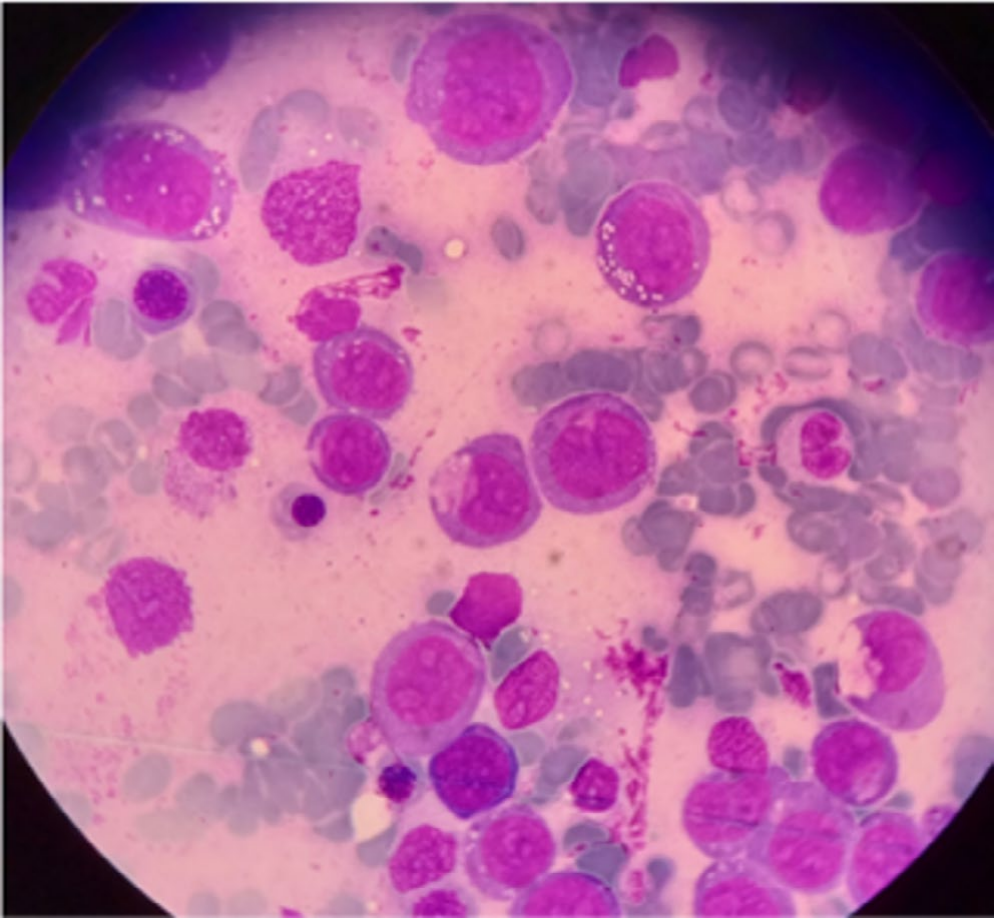
Bone marrow aspirate showing numerous large immature mononuclear cells with convoluted nuclei, prominent nucleoli, multiple vacuoles, and basophilic cytoplasm. Morphology is consistent with monoblasts and supports a diagnosis of AML with monocytic differentiation (e.g., AML‐M5). Wright‐Giemsa stain, magnification: 1000×.

Secondary causes of CA‐mediated hemolysis were investigated with mycoplasma and EBV serology, upper gastrointestinal endoscopy, colonoscopy, chest computed tomography (CT) scan, abdominopelvic magnetic resonance imaging (MRI), flow cytometry, and biopsy of bone marrow. The flow cytometry of the bone marrow sample had poor quality because of agglutination but showed 13% blasts, 37% monocytes, 40% neutrophils, and 10% lymphocytes. In the blast gate, more than 50% of the blasts were CD117, CD16, and CD64 positive, while in the monocyte gate, more than 65% of cells were myeloperoxidase, CD13, CD33, CD64, CD14, and CD56 positive. Bone marrow histology revealed that more than 50% of nuclear cells were monoblasts (Figure 2) that were CD68, CD117, CD56, and myeloperoxidase positive in immunohistochemical staining. All other mentioned surveys were negative for anything of interest. Acute myeloid leukemia (AML) M4 or M5 was the final diagnosis. The bone marrow specimen was sent to another center for a second opinion, which confirmed the diagnosis.

Treatment with 5‐azacytidine, with a dose of 100 mg daily for 7 days, and Venetoclax with an escalating dose up to 400 mg daily was started (based on NCCN guideline for AML therapy, version 1.2023) [15]. Rituximab injection was not repeated at all. Tumor lysis syndrome did not occur. The symptoms decreased gradually, and jaundice was resolved. The laboratory tests improved, and the Hb level became stable (Table 1). The patient was discharged on day 33. Venetoclax 400 mg daily was continued.

**TABLE 1:** Laboratory test results of patient during first admission.

Days post‐admission	Day 0	Day 7	Day 12	Day 18	Day 33
WBC (10^3^/μL)	15.5	20	—	1.1	2.6
RBC (10^6^/μL)	2.12	2.21	—	3.06	3.32
Hb (g/dL)	9	6.7	—	7.8	9.6
MCV (fL)	102.8	100	—	87.9	90.7
Platelet (10^3^/μL)	132	156	—	89	187
LDH (U/L)	1980	1410	—	726	663
Creatinine (mg/dL)	1.4	1.5	—	1.5	1.3
Uric acid (mg/dL)	6	—	—	6.4	4.6
AST (U/L)	56	69	—	—	21
ALT (U/L)	62	29	—	—	13
ALP (U/L)	284	198	—	—	157
Bilirubin (direct/total) (mg/dL)	2.3/5.2	0.9/2.2	1.1/2.7	—	—

Abbreviations: ALP, alkaline phosphatase; ALT, alanine aminotransferase; AST, aspartate aminotransferase; em dash (—), not assessed; Hb, hemoglobin level; LDH, lactate dehydrogenase; MCV, mean corpuscular volume; RBC, red blood cell; WBC, white blood cell.

Seven days after discharge, his WBC count was 3200 per cubic millimeter, Hb was 10.2 g/dL, and platelet count was 204,000 per cubic millimeter. One week later, while feeling progressive weakness, he presented with a WBC count of 18,800 per cubic millimeter. He was referred to our hospital for further evaluation and chemotherapy. A day later, he was admitted to the emergency ward while he was unable to stand due to weakness. His WBC was 170,400 per cubic millimeter. Hyperleukocytosis was proven to be due to monocytic leukemia, but there were no clues of hemolysis. Treatment adherence was approved subjectively and objectively, counting the number of pills taken. Unfortunately, he died because of leukostasis in hepatic circulation and liver failure on this admission.

## 4 Discussion

Our knowledge about CA‐mediated hemolytic anemia has improved in the past two decades. Today, we know that CAD is an independent bone marrow disease, called cold agglutination‐mediated bone marrow disease, and is distinct from other lymphoproliferative disorders. Secondary CA‐mediated hemolytic anemia is called CAS [1, 16].

Our patient had a medical history of red urine passage and blood sample clotting, and laboratory tests indicated the presence of CA autoantibodies and intravascular hemolysis. Similar to many cases of CAD, the patient does not show any cold‐induced symptoms, such as acrocyanosis or Raynaud phenomenon, which are not necessary for diagnosis. Although the direct bilirubin level was more than 15% of the total bilirubin, some degree of liver dysfunction was observed in the patient based on elevated liver enzymes, which can justify the rise of direct bilirubin despite the presence of hemolysis. Our patient suffered from a monocytic type of AML. Monocytes can infiltrate the liver and disturb hepatocyte function. A CA titer of 1/64 confirms the presence of CA. Although a major component of CA‐mediated hemolytic anemia is extravascular, sometimes the C3b complement activates C5, proceeding to major attack complex formation and leading to intravascular hemolysis. For the diagnosis and evaluation of CAD, a positive direct agglutination test and an agglutinin titer of 1/64 are at least required. The hemolysis degree is not dependent on the CA titer but on its thermal amplitude [2]. A negative direct antiglobulin test (DAT) is common in our region's laboratory and has already been seen in confirmed cases of CAD. This is probably due to a technical problem. The Anti‐human globulin (AHG) reagent used in our center was anti‐IgG AHG, while cold agglutinins are most often positive using anti‐C3d AHG reagents. Some other causes of false‐negative results are the low quantity of antibodies on RBCs, the improper temperature at which the test is carried out, and inappropriate antigen: antibody ratio and elution of low‐avidity antibodies from the coated RBCs during the washing process [17]. It is important to note that the specimen collected for testing agglutination should be stored at 37°C–38°C after collection.

IgM can be monoclonal or polyclonal. Similar to our patient, polyclonal IgM is most commonly seen in post‐infectious CA but also in all case reports of CAS associated with a malignancy, as mentioned earlier [10, 11, 12]. A history of fever a few days before admission may suggest an infection as the cause of CAS, which, of course, cannot be ruled out, but there are no other signs and symptoms of infection. Conversely, an infection could exacerbate previous CAS because the patient was symptomatic a few months before admission. The identification of the immunoglobulin class is not required for diagnosis, but the response to therapy is somehow dependent on the IG class.

This case emphasizes the necessity of considering underlying hematologic malignancies in individuals with CA‐mediated hemolytic anemia and of bone marrow sampling in these cases. Furthermore, it illustrates the challenges involved in diagnosing CAS linked with malignancies. Our case had the monocytic type of acute leukemia. We do not know the significance of this finding, but this should be in mind for future cases. As a conclusion, we have reported the first case of CAS associated with AML.

## Author Contributions

**Mohsen Vakili Sadeghi:** conceptualization, data curation, funding acquisition, investigation, project administration, resources, validation, writing – original draft, writing – review and editing. **Zeinab Vosough:** conceptualization, investigation, resources, validation, visualization, writing – original draft, writing – review and editing. **Davoud Jahansouz:** conceptualization, investigation, methodology, writing – original draft, writing – review and editing.

## Disclosure

Patient perspective: The patient reported a vast improvement in symptoms and asthenia. He was content with the medications after discharge and reported no major side effects.

## Ethics Statement

As a mandatory requirement in our country, this study was approved by the Babol University of Medical Sciences Ethics Committee under the code: IR.MUBABOL.HRI.REC.1403.037.

## Consent

Written informed consent was obtained from the patient to publish this report by the journal's patient consent policy.

## Conflicts of Interest

The authors declare no conflicts of interest.

## Data Availability

All de‐identified hospital records and paraclinical results are available upon request.
